# Social Network Analysis in Healthcare Settings: A Systematic Scoping Review

**DOI:** 10.1371/journal.pone.0041911

**Published:** 2012-08-03

**Authors:** Duncan Chambers, Paul Wilson, Carl Thompson, Melissa Harden

**Affiliations:** 1 Centre for Reviews and Dissemination, University of York, York, United Kingdom; 2 Department of Health Sciences, University of York, York, United Kingdom; University of New South Wales, Australia

## Abstract

**Background:**

Social network analysis (SNA) has been widely used across a range of disciplines but is most commonly applied to help improve the effectiveness and efficiency of decision making processes in commercial organisations. We are utilising SNA to inform the development and implementation of tailored behaviour-change interventions to improve the uptake of evidence into practice in the English National Health Service. To inform this work, we conducted a systematic scoping review to identify and evaluate the use of SNA as part of an intervention to support the implementation of change in healthcare settings.

**Methods and Findings:**

We searched ten bibliographic databases to October 2011. We also searched reference lists, hand searched selected journals and websites, and contacted experts in the field. To be eligible for the review, studies had to describe and report the results of an SNA performed with healthcare professionals (e.g. doctors, nurses, pharmacists, radiographers etc.) and others involved in their professional social networks. We included 52 completed studies, reported in 62 publications. Almost all of the studies were limited to cross sectional descriptions of networks; only one involved using the results of the SNA as part of an intervention to change practice.

**Conclusions:**

We found very little evidence for the potential of SNA being realised in healthcare settings. However, it seems unlikely that networks are less important in healthcare than other settings. Future research should seek to go beyond the merely descriptive to implement and evaluate SNA-based interventions.

## Introduction

Diffusion of innovations theory provides a framework for explaining how new ideas and practices spread within a social system [1]. In the UK, there has been renewed interest in the application of this theory to health care, due largely to concerns about the lack of uptake, and translation into practice, of knowledge on the effects of interventions in health care. Research funded by the NIHR (National Institute for Health Research) Service Delivery and Organisation Programme [2] and more recently the development of NIHR Collaborations for Leadership in Applied Health Research and Care (CLAHRCs), has refocused attention on the role of social interactions and networks in the ability of health service organisations to identify and exploit knowledge from outside the National Health Service (NHS).

Social network analysis (SNA) offers a means of mapping and exposing the hidden channels of communication and information flow, collaboration and disconnects between people in strategically important groups within an organisation [3], [4], [5], [6]. Rather than focusing solely on the strength of individual relationships, it explores the types of relationships that condition communication and learning. Social network analysis has been widely used across a range of disciplines but is most commonly applied to help improve the effectiveness and efficiency of decision making processes in commercial organisations. It does have some tradition of use in diffusion research [7], [8].

As part of the NIHR CLAHRC for Leeds, York and Bradford, we are utilising SNA to inform the development and implement of tailored behaviour-change interventions. These interventions are aimed at increasing the translation of research-based findings into local practice [9]. Our hope is that by taking a network perspective we will be able to identify, target and support those relationships and collaborations that generate better uptake and utilisation of knowledge. To inform this work, we have conducted this systematic scoping review of SNA studies conducted in a healthcare setting.

Our primary objective was to evaluate the use of SNA as part of an intervention to support the implementation of change in healthcare organisations. A secondary objective was to identify and describe studies that report the results of an SNA undertaken in a healthcare setting: and attempt to assess what they tell us about the role and influence of social networks in healthcare organisations.

## Methods

The review was carried out in accordance with a protocol developed in advance (File S1). The PRISMA (Preferred Reporting Items for Systematic Reviews and Meta-Analyses) checklist for this paper is presented as File S2.

### Literature Search

The literature search aimed to systematically identify social network analyses of healthcare professionals in any healthcare setting. A broad search strategy was initially developed on MEDLINE (OvidSP) using free text terms, synonyms and subject headings relating to social networks and methods used to investigate them. The strategy consisted of the main term social networks, various terms relating to the methods used to analyse or measure social networks, such as sociometrics, sociograms, sociomaps, and named software commonly used in social network analysis e.g. UCINET, NetDraw. In addition, the subject headings interprofessional relations, interdisciplinary communication and physician-nurse relationships were included in the strategy. The search strategy was adapted for use in the other databases searched.

The following databases were searched from 1950 to October 2011: MEDLINE and MEDLINE In-Process & Other Non-Indexed Citations; EMBASE; PsycINFO; Health Management Information Consortium (HMIC); the Cochrane Library (Cochrane Database of Systematic Reviews, Database of Abstracts of Reviews of Effects, Cochrane Central Register of Controlled Trials, Cochrane Methodology Register and Health Technology Assessment Database); CINAHL; Business Source Premier; Social Science Citation Index; Conference Proceedings Citation Index- Social Science & Humanities; and ASSIA. As social network analysis developed from 1950’s onwards, retrieval of studies was restricted to those published after 1950. No language restrictions or study design filters were applied to the search strategy. Further details of the database search strategy can be found in File S3.

The reference lists of relevant reviews and guidelines and included studies were checked for further potentially relevant studies. We also searched the website of the International Network for Social Network analysis (www.insna.org), including linked sites and the contents of the journal *Connections*. We hand searched the journals *Social Networks* and *Implementation Science* and contacted experts in the field with a view to identifying additional studies. Records were managed within an Endnote library (Endnote version X3).

### Inclusion and Exclusion Criteria

Titles and abstracts of records identified by the searches were assessed by two authors (DC and PW) independently. Full-text copies of any items thought to potentially meet the review inclusion criteria were obtained and assessed against the review inclusion criteria by the same two authors. Disagreements were resolved by consensus or by reference to a third author (CT).

To be eligible for the review, studies had to describe and report the results of an SNA performed with healthcare professionals (e.g. doctors, nurses, pharmacists, radiographers etc.) and others involved in their professional social networks (e.g. administrative, support and secretarial staff) in any healthcare setting. Those that went on to report on the use of the results of the SNA as part of an intervention to change some aspect of policy or practice were classified as level I studies. Studies that described the existing social networks in the organisation without reporting any follow-up action or its results were classified as level II studies.

Randomised and non-randomised controlled trials, controlled before and after studies and interrupted time series studies were eligible for inclusion as level I studies. Eligible comparisons were between organisations (SNA performed and used vs. no SNA) or within an organisation (before vs. after SNA performed and used). Level II studies could be of any design and did not need to have a comparator.

Outcomes of interest were any measure of the performance of a healthcare organisation or of individuals within it. Studies that used changes to social networks (measured by a follow-up SNA) as outcome measures were also eligible. Level II studies could have properties of the social network as outcomes.

Studies that used questionnaires or interviews to identify ‘opinion leaders’ but did not conduct an SNA were excluded, as were studies of patients’ and carers’ social networks.

### Quality Assessment and Data Extraction

Data on settings, participants, methods of data collection, SNA methods and results/conclusions were extracted from study reports by one author and checked by another. Separate data extraction sheets were developed and piloted for level I and level II studies. Quality (risk of bias) of level I studies was assessed by two authors (DC and PW) independently using the criteria of the Cochrane EPOC (Effective Practice and Organisation of Care) Group. Disagreements were resolved by discussion.

### Data Synthesis

Heterogeneity of settings, interventions and outcomes precluded meta-analysis. We therefore performed a narrative synthesis of the included studies. Level I and level II studies were considered separately. Limited outcome data meant that the synthesis of level II studies was descriptive.

## Results

**Figure 1:**
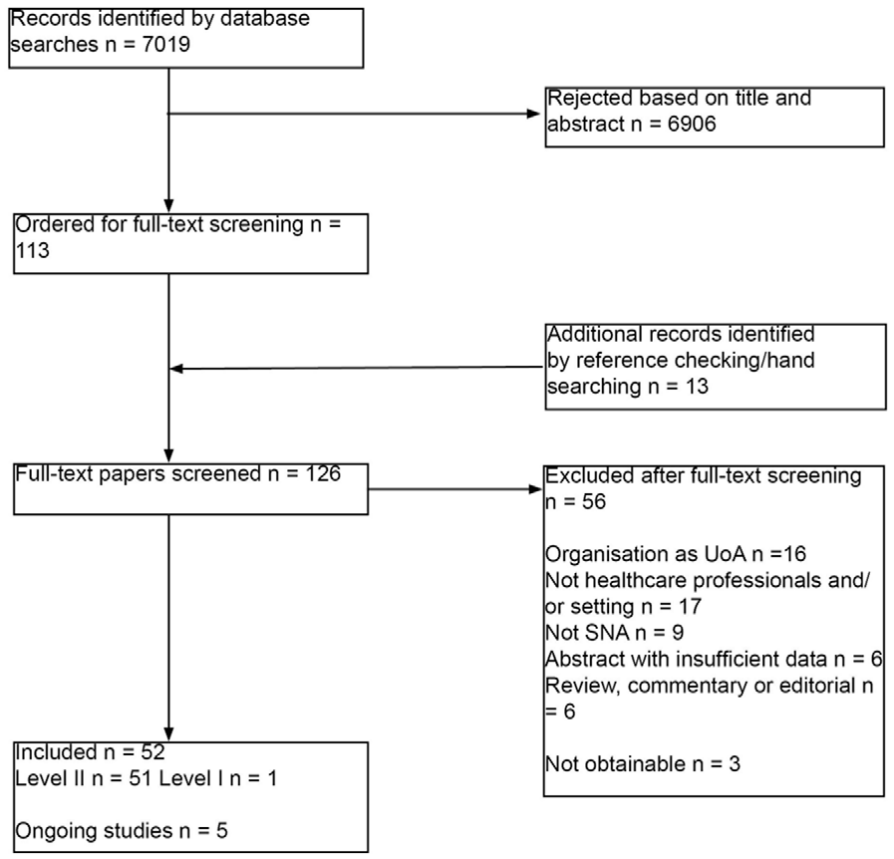
Flow chart of studies through the review process.

The study selection process is summarised in Figure 1. In total we included 52 completed studies, reported in 62 publications, of which only one was a level I study (i.e. it reported on a SNA in a healthcare setting and included use of the results as part of an intervention to change policy or practice) [10]. One ongoing study with no results available at the time of writing but with a published protocol was also considered as a potential level I study [11]. Fifty-one studies (61 publications) were classified as level II studies, i.e. they described or reported a SNA conducted in a healthcare setting but without reporting any follow-up action or its results. The shortage of level I studies and the large number of level II studies with only social network outcomes reported meant that we had to resort to a largely descriptive synthesis of the studies.

### Level I Study

In the only level I study found, Anderson et al. [10] conducted a quasi-experimental study using hospital services (departments) as the unit of analysis. The intervention used staff members identified (by SNA) as “educationally influential” to increase use of personal order sets (i.e. the ability to speed up ordering of drugs and tests by specifying in advance those that the doctors frequently order for their patients) within a hospital information system. Hospital data were used to construct binary consultation networks (i.e., who consulted who) among doctors in each service. Hierarchical clustering was used to generate groups of doctors with similar consultation patterns. Influential doctors (one per group) were identified based on measures of prestige in the consultation network. The influential doctors received educational outreach about the advantages of personal order sets. Control services received no intervention. There were 109 doctors in the experimental group and 231 in the control group but the number of educationally influential doctors was not reported. Quality assessment revealed a number of design limitations including non-random assignment and differences between experimental and control groups at baseline (see Table S1). This study was classified as a level I study rather than an opinion leader study because the influential physicians were identified based on their position in the social network rather than on peer nominations.

### Level II Studies

Selected characteristics of included studies are reported in Tables 1, 2, 3. The largest group of included studies (24) were conducted in the USA. There were 13 studies from European countries (excluding the UK), five from Canada, five from Australia and only three [12], [13], [14], [15] from the UK. Only one of the included studies was conducted in a low or middle-income country [16]. Participants who supplied social network data were doctors (18 studies), teams or mixed groups of health professionals (17 studies), nurses (nine studies) or other health professionals including administrators, emergency planners and policy makers (seven studies). Over half of the studies (32) were conducted in secondary or tertiary care settings, nine in primary care and only five involved both primary and secondary care.

**Table 1:** Summary of included level II studies: primary care settings.

Reference	Country	Participants	Data collection	Main findings and theme(s)
Scott 2005 [18]	USA	Doctors, nurses, admin staff	Observational	SNA is a useful tool for quantitative analysis of the complex interaction patterns represented by primary care practices. Themes: Decision making, communication
Zheng 2007 [47] 2010 [48]	USA	Doctors	Survey	Social influence seems to be mainly conveyed through interactions with personal friends rather than interactions in professional settings. Themes: Diffusion of innovations, Social influence
Keating2007 [49]	USA	Doctors	Survey	Physicians obtain information from colleagues with greater expertise and experience as well as colleagues who were accessible based on location and schedule. It may be possible to organize practices to promote more rapid dissemination of high-quality evidence-based medicine within primary care settings. Themes: Social influence
Chung2007, 2010[50], [51]	Australia	Doctors	Survey	Understanding of how network structure inter-relates with technology and its adopters may prove beneficial in increasing ICT uptake at the organizational (macro) and individual (micro) levels. Themes: Information sharing behaviour; diffusion of innovations
Fattore2009 [52]	Italy	GPs	Survey	In GP collaborative arrangements, the social influence mechanism (measured by network homogeneity) is more relevant than the social capital mechanism (measured by network centrality) for influencing prescribing behaviour. Themes: Prescribing behaviour/social influence
Martinez Arino 2009 [25]	Spain	Nurses; doctors; auxiliaries;social worker; physiotherapist;nurse manager (matron),admin staff	Survey	The professional support network was denser than the personal one and did not correspond exactly to the official structure. Friendships were mainly between members of similar professions. Themes: Communication/interpersonal relationships
Quinlan2010 [24]	Canada	Nurse practitioners	Survey	Clinical teams could use this methodology to evaluate their own clinical decisions and promote discussion within the team, thereby further enhancing mutual understanding within the team. Themes: Decision making
Wensing 2010 [40]	Netherlands	GPs, nurses, assistants	Survey	Further research is needed to refine the measure of information networks and to test the impact of network characteristics on the uptake of innovations. Themes: Diffusion of innovations; information sharing
Bradley2011 [15]	UK	GPs, community pharmacists	Survey	The findings suggest differences in GP and community pharmacist perceptions of the existence of contacts and also suggest greater familiarity between GPs and pharmacists in smaller geographical areas. Themes: Service provision/organisation

**Table 2:** Summary of included level II studies: secondary or tertiary care settings.

Reference	Country	Participants	Data collection	Main findings and theme(s)
Anderson 1985[53] 2002 [54]	USA	Doctors	Survey	Social network techniques can be effectively used to identify physicians who play different roles in the diffusion and adoption of new medical technologies. The results demonstrate the importance of peer influence in communicating information about the availability and efficacy of new practices and procedures and in validating their use in clinical settings. Themes: Diffusion of innovations, social influence
Anderson 1987[55] (also reportedin Anderson2002 [54])	USA	Doctors, surgeons	Survey	Physicians’ clinical patterns are influenced by a multitude of factors, one of the most important of which is their peers. The position of physicians in the consultation network significantly influenced their adoption and utilisation of new computer technology. Themes: Diffusion of innovations, social influence
Hiscott 1989 [39]	Canada	Psychiatric nurses,nursing assistants, others	Survey	The analysis reveals the importance of close co-worker networks for the discussion and potential resolution of job-related problems. Themes: Social support
Cott 1997 [56]	Canada	Nurses, health care assistants, doctors,other staff	Survey	While teamwork may be increasing the participation in decision-making by health professionals other than medicine, its effects are limited to a group of higher status professionals. The structure of the team acts to reproduce and perpetuate control of the division of labour within health care teams. Themes: Service provision/organisation, team structure
West 1999 [14], 2005 [13]	UK	Directors of Nursing, Medical Directors	Survey	Gaps in the network of informal ties will impede the dissemination of information and the spread of social influence between DNs and MDs. Dissemination and influence strategies that take features of the social structure into account are more likely to be successful. Themes: Social influence
MacPhee 2000[26]	USA	Nurses	Survey	Workplace networks are important to both types of nurses (flexible vs. traditional schedules). Nurses on flex schedules may form less social attachments in order to manage the increased demands of moving among multiple units. Themes: Social support
MacPhee 2002[27]	USA	Nurses	Survey	Nursing managers play significant roles as key members of rural nurses’ work place social support networks. Themes: Social support
Ankem 2003 [57]	USA	Radiologists	Survey	Conferences are important for creating early awareness, while interaction with colleagues is the most important factor in stimulating use of an innovation among later adopters. Among colleagues, opinion leaders in non academic hospitals may be more influential than individuals in the academic community. Themes: Diffusion of innovations; information seeking
Kravitz 2003 [58]	USA	Doctors, Midwives, Obstetricians	Survey	Simply identifying opinion leaders is of little avail if they are reluctant to endorse the innovations being introduced. A major question for future research is whether opinion leaders who are initially sceptical of desired clinical policies can be persuaded to embrace new approaches and bring their fellow clinicians along. Themes: Social influence; implementation/behaviour change
Pappas 2003,2004 [59], [60]	USA	Managers	Survey	Middle managers’ strategic knowledge and the prevailing social structure of the hospital interact to help bring about organisational change Themes: Organisational change, decision making
Tagliaventi 2006 [22]	Italy	Doctors, Radiotherapy Technicians, Medical Physicists, Nurses	Survey, Data analysis	Working side-by-side and having common organizational values are important bases for knowledge transfer between professional groups which belong to different networks of practice. Themes: Information sharing behaviour
Curran 2006,2007 [21], [61]	Canada	Doctors, nurses	Data analysis	Content analysis indicated that an online discussion forum could be useful for seeking various categories of knowledge across a range of content topics. SNA showed that online medium stimulates more knowledge seeking opportunities and interactions than offline and that a limited number of participants actively reach out in both networks. Themes: Service provision/organisation, communication
Creswick 2007, 2010 [62], [63]	Australia	Doctors, nurses, allied health professionals	Survey	Clinical staff tend to seek medication advice from members of their own profession, but some key individuals are used as sources of advice by all professional groups. Themes: Medication advice-seeking; social influence
Vanderveen 2007 [37]	USA	Surgeons	Survey	Surgical oncologists and university-based surgeons play key educational roles in disseminating new cancer treatments. Themes: Diffusion of innovations, social influence
Benham-Hutchins 2008 [64],2008 [65],2010 [66]	USA	Doctors, nurses, pharmacists, socialworker	Survey	The results provide a foundation for future research into how network structure and communication principles can be used to design health information technology that complements the information gathering and dissemination behaviour of health professionals involved in patient transfers. Themes: Communication/Service provision/organisation
Heiligers 2008[67]	Netherlands	Doctors	Survey	Part-time doctors do not aim to limit the size of their networks. The authors suggested that this could be because they want to stay in direct contact with all colleagues to prevent communication errors. Themes: Service provision/organisation/part-time working
Rangachari 2008 [30]	USA	Administrators	Survey	To improve hospital coding performance, it is important to co-ordinate knowledge exchange across physician and coding subgroups and connect these subgroups with the external environment. Themes: Service provision/organisation, information management
Barrera 2009 [17]	Netherlands	Nurses, students, support staff	Observation, Data analysis	Management could develop a policy for the appointment of nurses to the supervision of students, aiming at reducing the spread of distrust. Themes: Social support
Baumgart 2009 [20]	Germany	Nurses, anaesthetists, surgeons, assistants	Process logs	Use of mixed methods allowed a deeper understanding of the OR work context and its influence on outpatient OR processes. Themes: Service provision/organisation
Creswick 2009 [29]	Australia	Doctors, nurses, allied health professionals, administrative staff, ward assistants	Survey	SNA can provide insights of potential benefit to emergency department staff, their leaders, policymakers and researchers. Training in cross-disciplinary communication and interaction may be beneficial. Themes: Service provision/organisation; communication
Lurie 2009 [33]	USA	Doctors, residents, nurses and pharmacist	Survey	SNA provides indices of team functioning that could be used as predictor variables in studies of quality of care. Themes: Service provision/organisation/team culture
Samarth 2009 [34]	USA	Nurses, Operations Assistant	Survey	The design and performance of social networks is an important factor in improving process efficiencies within hospital organizations. Workflow redesign and implementation over an integrated IT backbone has to complement social network design in order to achieve an efficient integrated healthcare delivery system. Themes: Service provision/organisation, communication
Boyer 2010 [28]	France	Doctors, nurses, psychologists, social workers, administrators	Survey	SNA is useful for examining relationships in hospital organisations. Quantitative measurements allow comparisons across time which could be useful for evaluating the effects of interventions. Specific multidisciplinary management training may help different professional groups to work together. Themes: Service provision/organisation
Walton 2010 [19]	Canada	Doctors, medical students	Observational	In-patient rounds may not always fulfil their educational potential. The authors recommended that the order of patient discussion should be planned to highlight specific points and teaching staff should ensure that all team members are actively engaged in the process. Themes: Medical education
Menchik 2010 [68]	USA	Doctors	Survey	Differences between doctors at higher and lower prestige hospitals reflect their different priorities and roles in the production and diffusion of new research knowledge. Themes: Research utilisation
Myers 2010 [69]	USA	Nurses, nurse aides	Survey	The findings support a theoretical framework suggesting that patterns of relationships based on informal social status may contribute to differences in injury risk among individuals with the same job title. Themes: Service provision/organisation/occupational health
Jippes 2010 [38]	Netherlands	Gynaecologists and Paediatricians	Survey	Strong and weak ties within social networks seem to be more important than training and education for the diffusion of structured feedback. Themes: Medical education/diffusion of innovations
Nair 2010 [23]	USA	Doctors	Survey	This research adds to the literature that documents peer effects using individual consumer-level data. Opinion leader identification and targeting is of key importance to the pharma company in terms of increasing prescribing behaviour generally. Themes: Social influence, Prescribing behaviour, Implementation/behaviour change
Rangachari 2010 [32]	USA	Medical intensive care unit staff	Survey	The communication network structure indicated minimal interaction across professional subgroups and hierarchical levels. Analysis of communication content indicated that mainly explicit knowledge on general infection topics was being exchanged, rather than tacit knowledge on specific infection prevention practices. Themes: Service provision/organisation; implementation/behaviour change
Landim 2010 [16]	Brazil	Members of haematology nursing team	Survey	Analysing the network of personal relationships involving members of a team provides data that can be used to enable the team to work together more effectively. Themes: Social support; service provision/organisation
Sykes 2011 [70]	USA	Doctors	Survey	Those implementing electronic medical record (EMR) systems need to be aware that the better-connected doctors, who also tend to be better performing in terms of patient satisfaction, will tend to use EMR systems less than those with less central network positions. Administrators and other stakeholders can use this information to develop interventions to increase doctors’ support and use of the system. Themes: Diffusion of innovations
Mascia 2011 [71], [72]	Italy	Doctors	Survey	Healthcare organisations are likely to contain separate clusters of doctors whose members are highly similar. The authors suggested that organisational interventions are needed to encourage heterophily in settings where multidisciplinary co-operation is required to provide effective health care. The cohesion associated with constrained social networks may hamper rather than support the diffusion of new information within professional groups. Themes: Diffusion of innovations; social influence
Zappa 2011 [73]	Italy	Doctors	Survey	Interaction between doctors can be a powerful tool for the diffusion of innovations but needs to be supported by other strategies, particularly communication campaigns. Themes: Diffusion of innovations; social influence

**Table 3:** Summary of included level II studies: other/mixed settings.

Reference	Country	Participants	Setting	Data collection	Main findings and theme(s)
Coleman 1957 [7]	USA	Doctors	Primary and Secondary care	Survey	A doctor will be influenced more by what his colleagues say and do in uncertain situations (e.g. when a drug is new), whenever and where ever they may occur, than in clear cut situations. Themes: Social influence, Prescribing behaviour, Diffusion of innovations
Winick 1961 [35]	USA	Doctors	Primary and Secondary care	Survey	The findings suggest the possibility that the large city pattern of diffusion of innovation of a new drug may be dependent on other methods of communication than has been previously found in smaller communities, i.e. personal communication may be less important. Themes: Social influence, Prescribing behaviour, Diffusion of innovations
Grimshaw 2006 [12]	UK	GPs, practice nurses, practice managers; hospital doctors, surgeons, and nursing staff; gynaecologists and oncologists	Primary and secondary care	Survey	The feasibility of identifying opinion leaders using a standard sociometric instrument varies across different professional groups and settings. The more specialised the group, the more recruitment of opinion leaders may be a useful strategy for influencing their behaviour. Themes: Behaviour change/opinion leaders
Lewis 2006 [74]	Australia	Policy makers (roles not stated)	Health policy	Survey	There are few signs that the power of medicine to shape the health policy process has been greatly diminished in Victoria. Medical expertise connects actors through ties of association, making it difficult for actors with other resources and different knowledge to be considered influential. SNA techniques provide novel and useful means for understanding the structures of influence which impact on the health policy process. Themes: Social influence
Harris 2007 [75]	USA	Emergency planners	Public health	Survey	Missouri public health emergency planners maintain large and varied networks but there are opportunities for strengthening existing ties and seeking additional connections. Themes: Service provision/organisation
Lower 2010 [76]	Australia	Nurses, GPs, ENT specialists, occupational health, other	Primary and community care	Survey	Social network analysis can assist in defining hearing health networks and can be used to highlight potential actions to strengthen networks. Themes: Service provision/organisation
Iyengar 2011 [77]	USA	Doctors	Primary and secondary care?	Survey	Findings from an SNA of prescribers of a new drug suggest ways to both increase theoretical understanding of social contagion and potentially to improve the effectiveness of network marketing. Themes: Diffusion of innovations; social influence
Van Beek 2011 [36]	Netherlands	Nurses	Community care	Survey	Communication and advice networks of nursing staff working in long-term care are relatively dense, reflecting the high level of co-operation needed to provide high-quality care. Networks are denser in smaller units and are influenced by staff members’ characteristics. Communication networks are also important for job satisfaction. Themes: Social influence; service provision/organisation
Wensing 2011 [78]	Netherlands	Various (health professionals treating patients with Parkinson’s disease)	Primary and secondary care	Survey	Network measures reflecting professional contacts showed relevant variation among health professionals. A larger caseload and a hospital affiliation were associated with stronger connections with other health professionals. Themes: Service provision/organisation; social influence

Social network data were collected by surveys in the great majority of studies. Observation of social interactions was used in three studies [17], [18], [19]. Use of process logs or other administrative data to construct social networks was reported in four studies [17], [20], [21], [22].

Almost all of the studies used a comparative or non-comparative cross-sectional design, with data collected at a single time point. The exceptions are briefly discussed below.

Scott et al. used data collected as part of a randomised trial to demonstrate how SNA can be used to characterize and compare communication patterns in primary care practices [18]. Nair et al. [23] used data from a market research survey (including opinion leader data) and prescription data for a new drug to quantify the impact of social interactions and peer effects in the context of physicians’ prescription choices. A study by Barrera et al. of the development of trust among nurses and other staff in a dialysis department in a Dutch hospital used social network data collected at several time points over a 1-year period [17]. Finally, Baumgart et al. [20] investigated social networks among operating room staff before and after a change in layout. This study was not classified as a level I study because the SNA was descriptive and not used to inform the intervention (change in operating room layout).

The two most common areas of focus identified via a qualitative examination of the studies were explorations of social networks in relation to service provision and organisation (19 studies) and their those examining the role of social networks in the context of behaviour change (22 studies, including diffusion of innovations, opinion leaders and other aspects of social influence). Other areas of focus included decision-making [24], interpersonal relations, [25] information sharing behaviour [22] and social support [17], [26], [27].

Key findings of studies that looked at service provision and organisation included differences in actual and perceived nature of social networks among professionals from different disciplines and weakness of links across disciplines [28], [29], [30], [31], [32]. A number of studies recommended training or other measures to strengthen such links. The potential value of SNA to measure team function and use the information to improve working processes was another finding of studies in different settings [33], [34].

SNA has been used to study social influence on health professionals and particularly the diffusion of innovations since the 1950s [7], [35]. Studies included in the review reported on differences across settings, for example the increased importance of social networks in smaller groups [35], [36], and on the importance of particular groups (for example, university-based surgical oncologists in a cancer network [37]) in promoting adoption of new practices. Jippes et al. reported that social networks were more effective than training for disseminating a new structured feedback technique [38].

SNA can also reveal the networks used by health professionals for social support, with studies reporting on the importance of close ties with co-workers in a potentially stressful setting (psychiatric hospital) [39]; the differences between nurses working normal and flexible schedules [26]; and the potential of early social support to reduce distrust among nursing team members [17].

The studies varied from those that appeared to be applying the methodology of SNA in a healthcare setting [18], [40] to some that suggested the usefulness of SNA for understanding and possibly changing the structure and processes of healthcare organisations. However, the latter group stopped short of suggesting how this might be achieved. For example, Creswick et al. stated that the results of SNA ‘can provide insights of potential benefit to emergency department staff, their leaders, policymakers and researchers’ [29] but did not enlarge on what the benefits might be. Samarth et al. [34] suggested ways in which social networks might be redesigned to improve patient flow through a post-anaesthesia care unit in a US teaching hospital. The authors referred to plans for a future study that ‘tests the effects of reconfiguration of social network patterns’. This study would change their work from a level II to a level I study in our terminology.

In summary, the level II studies report the results of SNAs conducted in a variety of healthcare settings, mainly using survey data and a cross-sectional design. While some hint at the possibility of using the results to design or implement interventions to change policy or practice, the majority are purely descriptive in nature.

### Ongoing Studies

We identified five relevant ongoing studies, three of which are funded by the NIHR Service Delivery and Organisation programme as part of its knowledge mobilisation [41], [42] and models of service delivery [43] research themes. Sales et al. [11] have published a protocol for a study of the impact of social networks on knowledge transfer in long-term care facilities, specifically the uptake and use of feedback reports (monthly reports documenting processes of care linked to modifiable outcomes). The study is intended to contribute to the design of interventions using social networks to promote knowledge translation. As such it can be considered as a potential level I study. Bradley et al. [31] published a preliminary report of a study using SNA to study integrated working between general practitioners and community pharmacists in the UK. The authors state that the study has the potential to ‘ensure that future policy decisions related to integrated working are evidence based’. However, no further details of the study were available at the time of writing.

## Discussion

This systematic scoping review presents to our knowledge the most comprehensive overview of SNA studies conducted in a healthcare setting. Fifty-two completed studies met our inclusion criteria, with an additional five identified as ongoing.

However, our primary objective was to identify and evaluate the use of SNA as part of an intervention to support the implementation of change in healthcare organisations. What is striking is that nearly all the literature is descriptive in nature; and only one study has used the results of an SNA to bring about change, specifically to increase the use of personal order sets by physicians in a hospital information system.

The search strategy employed was deliberately broad, and we searched a number of relevant databases and other sources with no language or study design restrictions to reduce the chance that relevant studies were missed and to prevent language bias. As an aid to transparency, we have included the list of excluded studies as Table S2. However, we anticipate that there may be a grey literature of potentially relevant studies that our searches have been unable to access. For example, the report by Cunningham et al. discussed below [44] was not located by any of our systematic searches but by a preliminary less systematic internet search. In the commercial sector, SNA are often conducted to examine the effectiveness of internal/external communications and to inform the implementation of change management programmes. It is very likely that similar studies have been conducted in health care settings but also that the reports of such activity have not been made publicly available. This phenomenon represents a form of ‘publication bias’. There is potential for further research to examine the presence/magnitude of this literature in specific sectors, for example by a survey of relevant healthcare organisations.

The major limitation of the review reflects the limitations of the evidence base and the almost complete lack of studies involving SNA as part of an intervention. The use of sociometric questionnaires to identify opinion leaders appears commonplace [45], but without the execution of an SNA component, these studies would have been excluded from our review. The numerous level II studies included in the review mostly used a cross-sectional design with no comparator and hence tell us nothing about the effect of social networks and SNA on change over time. Some studies suggested the existence of distinct networks within an organisation, e.g. advice-seeking and social, although these categories may be imposed by researchers.

We have provided a descriptive synthesis of the level II studies, including some key findings, primarily as an aid to future research. The conclusions that can be drawn from this synthesis are limited by the fact that these represent a heterogeneous group of studies whose only real common factor is the use of SNA methods to describe social interaction in a variety of healthcare settings at one specific point in time.

The one included level I study does appear to show a change in uptake of personal order sets over time but as a single study it does not provide an adequate basis for drawing conclusions. Furthermore the study had some methodological weaknesses, including differences between groups at baseline and uncertainty over whether members of the control group could have been exposed to the intervention. A more robust design to test the effectiveness of SNA in identifying influential individuals would be to compare educational outreach to individuals identified by SNA versus outreach to a randomly chosen sample.

We found one published review that covered a similar but not identical topic. Cunningham et al. [44] systematically reviewed the literature on the social and professional networks of health professionals. Despite the arguably wider focus of their review, Cunningham et al. included fewer studies than we did (40 vs. 52). This may be in part because their search covered a more limited timeframe (1995–2009) and thus the earlier research on for example, diffusion of innovations through social networks was not included. Other differences between the two reviews reflect their different objectives. While we focused on the results of SNA in healthcare settings, Cunningham et al.’s research question related to how research on social and professional networks has been used to examine the effectiveness and sustainability of networks in relation to quality of care and safety.

Cunningham et al. identified a similar range of key topics/themes to those identified by our review, including understanding the structural relationships and social context of professionals or organisations (corresponding roughly to our categories of service provision/organisation, social influence and social support), information/knowledge exchange or advice seeking of health practitioners, communication and exchange of patient clinical and other information between practitioners or organisations (covered by our service provision/organisation topic) and influence of information sources on awareness and adoption of a new technology or innovations (diffusion of innovations).

There are many factors that could help to explain the lack of visible evidence for the potential of SNA being realised in healthcare settings. For example, there are likely to be more constraints on the organisation’s ability to make change in response to the results of an SNA in a healthcare setting compared with the commercial sector. Another factor could be the background of researchers who have used SNA in healthcare settings. We did not systematically examine this but many authors of studies included in the review appear to be ‘pure’ social science researchers rather than having a background in applied or implementation research. There could be scope for qualitative research into why researchers who have performed SNAs in healthcare organisations have in many cases stopped short of suggesting concrete follow-up of their research.

Research methods used by those undertaking SNAs in healthcare settings will also be influenced by the size of the organisation; for example, small organisations allow for personal administration of questionnaires with high response rates. This may not be feasible for larger organisations but potentially more use could be made of administrative data as done in a few of the studies included in our review.

Researchers using SNA need to decide where network boundaries should be placed as relevant networks may go far beyond the boundary of the respondent’s own organisation. Another issue is the extent to which findings can be transferred beyond the context of a particular study. This is particularly relevant to attempts to use SNA as part of an intervention to change policy or practice. The use of labels like ‘bounded’ and ‘unbounded’ to describe networks may not be adequate for helping others to understand one network and apply it to their own context. An underlying theory may be helpful for guiding the development and evaluation of interventions aimed at bringing about change [46]. Although there are many theories regarding the structure of networks within and between organisations [44], it is unclear to what extent the studies included in our review used theory to guide their exploration and analysis of healthcare social networks. We did not explore this issue because it was not part of our protocol and objectives but it could be a topic for further research.

There is currently an absence of evidence to demonstrate that using SNA can enable intelligent targeting of key relationships and collaborations to facilitate better uptake and utilisation of knowledge. Future studies involving SNA in healthcare should be designed with an intervention and comparator. SNAs can be either dependent or independent variables not divorced from any other intervention (independent variable) or measurement (dependent variable). There is a risk that SNA discourse and time may foster a separation from classical literature on attribution of change to causes and questions of bias; to avoid this, level I studies need to be adequately powered and designed with appropriate comparators.

In conclusion, we found very little evidence for the potential of SNA being realised in healthcare settings. However, it seems unlikely that networks are less important in healthcare than other settings. Future research should seek to go beyond the merely descriptive to implement and evaluate SNA-based interventions.

## Supporting Information

Table S1 **Quality assessment of level I study.**
(DOCX)

Table S2 **Table of excluded studies.**
(DOCX)

File S1 **Review protocol.**
(DOCX)

File S2 **PRISMA checklist.**
(DOC)

File S3 **Details of search strategy.**
(DOCX)
