# Supplementary material for: Social Network Analysis in Healthcare Settings: A Systematic Scoping Review
Source: PLoS One. 2012 Aug 3;7(8):e41911. doi: 10.1371/journal.pone.0041911 (PMC3411695; doi:10.1371/journal.pone.0041911)
Supplement: Table S1 — Quality assessment of level I study. (DOCX) [file pone.0041911.s001.docx]

**Table S1**

**Quality assessment of level I study**

| Reference | Anderson 1990[10] |
| --- | --- |
| Study design | Quasi-experimental design using hospital services (departments) as the unit of analysis |
| Authors’ objectives | To design, implement and evaluate a programme to change the procedure used by physicians to enter orders into a hospital information system (HIS). The intervention used staff members identified as (by social network analysis) educationally influential to influence practice behaviour. |
| Participants (health professionals) | Profession: Doctors  Clinical specialty: Experimental group: cardiovascular disease; general surgery; obstetrics and gynaecology; orthopaedic surgery. Control group: ten other hospital services (not specified)  Level of training: Not reported  Age: Not reported  Years since graduation or in practice: Not reported  Proportion of eligible professionals (or practices etc.) that participated in the study: Not reported |
| Participants (patients) | Not applicable |
| Participants (other) | None |
| Numbers included in study | Data were obtained for 109 doctors in the experimental group and 231 in the control group |
| Setting | Type of healthcare system: Private  Type of setting: Teaching hospital  Country: USA |
| SNA intervention | Objective(s) of the intervention (if not covered above): To identify influential doctors in each of the services in the experimental group  Type of SNA used: Hospital data were used to construct binary consultation networks (i.e., who consulted who) among doctors in each service. STRUCTURE, a hierarchical clustering program, was used to generate groups of doctors with similar consultation patterns. Influential doctors (one per group) were identified based on measures of prestige in the consultation network; participation in the hospital’s medical education programme; and/or participation on medical staff committees.  Are details available (e.g. copy of questionnaire)? Not applicable. It appears that all data were obtained from hospital information systems so no questionnaires were used (based on information in another paper[46])..  How many people received the questionnaire? Not applicable  How were participants selected? Not applicable  Response rate: Not applicable  How were results used? Doctors identified as influential were asked to participate in a project to increase use of personal order sets to enter orders (for tests, drugs etc.) into the hospital order system |
| Other implementation intervention | Type of intervention: Educational outreach  Objectives of intervention: To increase use of personal order sets as above.  How many people received the intervention? It appears that there were five influential doctors in cardiovascular disease and general surgery and three each in obstetrics and gynaecology and orthopaedic surgery (total 16) were identified as influential and received the intervention.  Brief details of intervention as applicable, e.g. group or individual, deliverer, timing, setting: Influential doctors were involved in two meetings with project staff. Before the first meeting, a questionnaire was used to determine the extent to which each doctor used the HIS. A second questionnaire addressed use of personal and departmental order sets.  At the first meeting, doctors were given data on their use of the system compared with others on their service and the hospital as a whole. Project staff discussed the advantages of using personal order sets. Project staff did not ask the doctors to engage in any special activities to promote personal order sets.  Six months later a second meeting was held with the influential doctors at which they were provided with information on the use of the HIS by house staff and order entry times and error rates with regular HIS pathways and personal order sets. A summary of perceptions of the advantages and disadvantages of personal order sets (developed after the first meetings) was used to review the value of the educational programme and reinforce its content.  Data on numbers of orders entered into the HIS using personal order sets were collected before the intervention; two months after the first meeting; and two months after the second meeting. Order entries by doctors, unit secretaries and physician assistants were analysed using multivariate analysis of variance with repeated measures over time. The experimental and control services were compared. |
| Control group | What did they receive? No intervention, standard care/practice or another intervention (specify): No intervention  Number in control group: 231 |
| Results | **Controlled studies**  Outcome: Mean number of medical orders entered per patient using personal order sets   \|  \| Doctors \| Secretaries \| Assistants \| \| --- \| --- \| --- \| --- \| \| Pre intervention \| Experimental: 2.61  Control: 0.42 \| Experimental: 2.60  Control: 1.83 \| Experimental: 1.04  Control: 0.11 \| \| After first meeting \| Experimental: 3.02  Control: 0.55 \| Experimental: 3.64  Control: 1.96 \| Experimental: 1.49  Control: 0.17 \| \| After second meeting \| Experimental: 3.25  Control: 0.52 \| Experimental: 3.31  Control: 1.63 \| Experimental: 1.76  Control: 0.13 \|   Significance: There were significant differences between the experimental and control groups and between people entering the orders (doctors vs. secretaries vs. assistants). The group by time interaction was significant, indicating that use of personal order sets increased over time in the experimental group but not in the control group. |
| Risk of Bias | 1. Allocation sequence adequately generated = No 2. Allocation adequately concealed = Yes 3. Baseline outcome measurements similar = Yes? (ANOVA used to adjust for differences between individuals) 4. Baseline characteristics similar = No 5. Incomplete outcome data adequately addressed = Yes 6. Knowledge of the allocated interventions adequately prevented during the study = Yes   Study adequately protected against contamination = Unclear (others could’ve been exposed to the intervention)   1. Study free from selective outcome reporting = Yes 2. Study free from other risks of bias = Yes |
