# Supplementary material for: Social Network Analysis in Healthcare Settings: A Systematic Scoping Review
Source: PLoS One. 2012 Aug 3;7(8):e41911. doi: 10.1371/journal.pone.0041911 (PMC3411695; doi:10.1371/journal.pone.0041911)
Supplement: Table S2 — Table of excluded studies. (DOCX) [file pone.0041911.s002.docx]

**Table S2**

**Table of excluded studies**

| **Reference** | **Main reason for exclusion** |
| --- | --- |
| **Papers from database search** |  |
| Kunster 2010[1] | Organisation as unit of analysis |
| Ramanadhan 2010[2] | Not healthcare professionals or setting |
| Leischow 2010[3] | Not healthcare setting |
| Ramanadhan 2010[4] | Not healthcare setting |
| Hossain 2010[5] | Not healthcare professionals or setting |
| Hall 2010[6] | Organisation as unit of analysis |
| Gundlapalli 2009[7] | Not focused on health professionals’ networks |
| Elwood 1983[8] | Discussion paper |
| Meltzer 2010[9] | Review/discussion paper |
| Polgreen 2010[10] | Not SNA |
| Ramanadhan 2009[11] | Not health professionals/setting |
| Moses 2009[12] | Not health professionals/setting |
| Bilge 2009[13] | Not health professionals |
| Ueno 2008[14] | Not primarily focused on health professionals’ networks; simulation study |
| Lewis 2008[15] | Organisation as unit of analysis |
| Fuller 2007[16] | Organisation as unit of analysis? |
| Thomas 2007[17] | Organisation as unit of analysis |
| Chanut 2005[18] | Not health professionals; |
| Boyer 2005[19] | Not health professionals; similar to #788 |
| Heikkinen 2005[20] | Not SNA |
| Heng 2005[21] | Not healthcare professionals (facilities managers) |
| Timpka 1996[22] | Not SNA |
| Mendel 2009[23] | Organisation as unit of analysis |
| Lamontagne 2010[24] | Abstract with limited results |
| Ladak 2010[25] | Abstract with limited results |
| Patterson 2010[26] | Abstract with limited results |
| Scott 2007[27] | General review, no specific results |
| Luque 2010[28] | Organisation as unit of analysis |
| Johanson 2000[29] | Not healthcare professionals/setting |
| Anderson 1991[30] | Not SNA |
| Provan 2004[31] | Organisation as unit of analysis |
| Fischbach 2010[32] | No methods or results for hospital |
| Lindholm 2003[33] | No SNA (general social questions) |
| **Additional orders** |  |
| Norman 2006[34] | Not healthcare setting (community of practice) |
| Brownson 2010[35] | Organisation as unit of analysis |
| Merrill 2008[36] | Research department, not healthcare setting |
| Gold 2008[37] | Organisation as unit of analysis |
| Bilge 2006[38] | No real data presented |
| Krauss 2004[39] | Organisation as unit of analysis |
| Chiu 2007[40] | Not health professionals (lay health educators) |
| Valente 2008[41] | Organisation as unit of analysis |
| Eisenberg 1996[42] | Organisation as unit of analysis |
| Tausig 1987[43] | Organisation as unit of analysis |
| **Additional handsearch papers** |  |
| Hirdes 1998[44] | Main focus is patients, also no relevant data |
| Wright 1995[45] | Organisation as unit of analysis |
| Parchman 2011[46] | Organisation as unit of analysis |
| **Update searches** |  |
| Goodwin 2010[47] | Editorial |
| Dunn 2011[48] | Review/discussion paper |
| Malin 2011[49] | Not SNA |
| Hripcsak 2011[50] | Not SNA |
| Effken 2011[51] | Editorial |
| Guldbrandsson 2010[52] | Abstract with limited results |
| Varda 2011[53] | Not SNA (simulation) |
| Christakis 2011[54] | Not health professionals/setting |
| **Update handsearch** |  |
| Palinkas 2011[55] | Not health professionals/setting |
| Doumit 2011[56] | Not SNA |

**References to excluded studies**

1. Kunster AK, Knorr C, Fegert JM, Ziegenhain U (2010) [Social network analysis of interdisciplinary cooperation and networking in early prevention and intervention. A pilot study]. Bundesgesundheitsblatt, Gesundheitsforschung, Gesundheitsschutz 53(11): 1134-1142.

2. Ramanadhan S, Wiecha JL, Gortmaker SL, Emmons KM, Viswanath K (2010) Informal training in staff networks to support dissemination of health promotion programs. American Journal of Health Promotion 25(1): 12-18.

3. Leischow SJ, Luke DA, Mueller N, Harris JK, Ponder P, et al. (2010) Mapping U.S. government tobacco control leadership: networked for success? Nicotine & Tobacco Research 12(9): 888-894.

4. Ramanadhan S, Kebede S, Mantopoulos J, Bradley EH (2010) Network-based social capital and capacity-building programs: an example from Ethiopia. Human Resources for Health 8(17.

5. Hossain L, Kuti M (2010) Disaster response preparedness coordination through social networks. Disasters 34(3): 755-786.

6. Hall JN, Moore S, Shiell A (2010) Assessing the congruence between perceived connectivity and network centrality measures specific to pandemic influenza preparedness in Alberta. BMC Public Health 10(124.

7. Gundlapalli A, Ma X, Benuzillo J, Pettey W, Greenberg R, et al. (2009) Social network analyses of patient-healthcare worker interactions: implications for disease transmission. AMIA Annual Symposium Proceedings: 213-217.

8. Elwood TW (1983) The application of social network theory to hospital cost containment. International Quarterly of Community Health Education 4(3): 183-190.

9. Meltzer D, Chung J, Khalili P, Marlow E, Arora V, et al. (2010) Exploring the use of social network methods in designing healthcare quality improvement teams. Social Science & Medicine 71(6): 1119-1130.

10. Polgreen PM, Tassier TL, Pemmaraju SV, Segre AM (2010) Prioritizing healthcare worker vaccinations on the basis of social network analysis. Infection Control and Hospital Epidemiology 31(9): 893-900.

11. Ramanadhan S, Wiecha JL, Emmons KM, Gortmaker SL, Viswanath K (2009) Extra-team connections for knowledge transfer between staff teams. Health Education Research 24(6): 967-976.

12. Moses AS, Skinner DH, Hicks E, O'Sullivan PS (2009) Developing an educator network: the effect of a teaching scholars program in the health professions on networking and productivity. Teaching and Learning in Medicine 21(3): 175-179.

13. Bilge U, Senol U, Saka O (2009) Informal social networks amongst administrative staff at a university hospital. In: Adlassnig KP, Blobel B, Mantas J, Masic I, editors. Medical Informatics in a United and Healthy Europe - Proceedings of MIE 2009. Amsterdam: IOS Press. pp. 282-286.

14. Ueno T, Masuda N (2008) Controlling nosocomial infection based on structure of hospital social networks. Journal of Theoretical Biology 254(3): 655-666.

15. Lewis JM, Baeza JI, Alexander D (2008) Partnerships in primary care in Australia: network structure, dynamics and sustainability. Social Science & Medicine 67(2): 280-291.

16. Fuller J, Kelly B, Sartore G, Fragar L, Tonna A, et al. (2007) Use of social network analysis to describe service links for farmers' mental health. Australian Journal of Rural Health 15(2): 99-106.

17. Thomas JC, Isler MR, Carter C, Torrone E (2007) An interagency network perspective on HIV prevention. Sexually Transmitted Diseases 34(2): 71-75.

18. Chanut C, Boyer L, Robitail S, Horte C, Jacqueme B, et al. (2005) [Applying social network analysis to the health system]. Sante Publique 17(3): 403-415.

19. Boyer L, Chanut C, Horte C, Mabriez JC, Auquier P (2005) [Analysis of transfer from one hospital to another in patients with myocardial infarction in the Provence-Alpes-Cote-d'Azur]. Annales de cardiologie et d'angéiologie 54(5): 233-240.

20. Heikkinen A, Puura K, Mattila K (2005) Improving health centre physicians' child-psychiatric networks. Scandinavian Journal of Primary Health Care 23(1): 26-27.

21. Heng HKS, McGeorge WD, Loosemore M (2005) Beyond strategy: exploring the brokerage role of facilities manager in hospitals. Journal of Health Organization and Management 19(1): 16-31.

22. Timpka T, Hallberg N (1996) Talking at work - professional advice-seeking at primary health care centres. Scandinavian Journal of Primary Health Care 14(3): 130-135.

23. Mendel P, Damberg CL, Sorbero MES, Varda DM, Farley DO (2009) The growth of partnerships to support patient safety practice adoption. Health Services Research 44(2, Part II): 717-738.

24. Lamontagne ME, Bonnie RS, Lavoie A (2010) The use of social network analysis in the study of neurotrauma networks. Brain Injury 24(3): 260-261.

25. Ladak S, Gillett J, Everest K (2010) Social network analysis as a tool for evaluating the collaborative potential within the international pediatric brain injury society: a baseline analysis. Brain Injury 24(3): 254-255.

26. Patterson D, Yealy DM, Krackhardt D, Abebe K, Weaver MD (2010) Variation in EMT team configuration and teammate familiarity. Clinical and Translational Science 3(2): S27.

27. Scott C, Hofmeyer A (2007) Networks and social capital: a relational approach to primary healthcare reform. Health Research Policy and Systems 5(9.

28. Luque J, Martinez Tyson D, Ji-Hyun L, Gwede C, Vadaparampil S, et al. (2010) Using social network analysis to evaluate community capacity building of a regional community cancer network. Journal of Community Psychology 38(5): 656-668.

29. Johanson J-E (2000) Formal structure and intra-organisational networks. An analysis in a combined social and health organisation in Finland. Scandinavian Journal of Management 16(3): 249-267.

30. Anderson JG (1991) Stress and burnout among nurses: a social network approach. Journal of Social Behavior and Personality 6(7): 251-272.

31. Provan KG, Veazie MA, Teufel-Shone N, Huddleston C (2004) Network analysis as a tool for assessing and building community capacity for provision of chronic disease services. Health Promotion Practice 5(2): 174-181.

32. Fischbach K, Gloor PA, Lassenius C, Olguin DO, Pentland A, et al. (2010) Analyzing the flow of knowledge with sociometric badges. Procedia Social and Behavioral Sciences 2(6389-6397.

33. Lindholm M, Dejin-Karlsson E, Ostergren PO, Uden G (2003) Nurse managers' professional networks, psychosocial resources and self-rated health. Journal of Advanced Nursing 42(5): 506-515.

34. Norman CD, Huerta T (2006) Knowledge transfer & exchange through social networks: building foundations for a community of practice within tobacco control. Implementation Science 1(20.

35. Brownson RC, Parra DC, Dauti M, Harris JK, Hallal PC, et al. (2010) Assembling the puzzle for promoting physical activity in Brazil: a social network analysis. Journal of Physical Activity and Health 7(Suppl. 2)(S242-252.

36. Merrill J, Hripcsak G (2008) Using social network analysis within a department of biomedical informatics to induce a discussion of academic communities of practice. Journal of the American Medical Informatics Association 15(6): 780-782.

37. Gold M, Doreian P, Taylor EF (2008) Understanding a collaborative effort to reduce racial and ethnic disparities in health care: contributions from social network analysis. Social Science & Medicine 67(6): 1018-1027.

38. Bilge U, Saka O (2006) Agent based simulations in healthcare. In: Hasman A, Reinhold H, van der Lei J, De Clercq E, Roger-France F, editors. Ubiquity: technologies for better health in aging societies Proceedings of MIE 2006. Amsterdam: IOS Press. pp. 699-704.

39. Krauss M, Mueller N, Luke D (2004) Interorganizational relationships within state tobacco control networks: a social network analysis. Preventing Chronic Disease 1(4): A08.

40. Chiu LF, West RM (2007) Health intervention in social context: understanding social networks and neighbourhood. Social Science & Medicine 65(9): 1915-1927.

41. Valente TW, Coronges KA, Stevens GD, Cousineau MR (2008) Collaboration and competition in a children's health initiative coalition: a network analysis. Evaluation and Program Planning 31(4): 392-402.

42. Eisenberg M, Swanson N (1996) Organizational network analysis as a tool for program evaluation. Evaluation and the Health Professions 19(4): 488-506.

43. Tausig M (1987) Detecting 'cracks' in mental health service systems: application of network analytic techniques. American Journal of Community Psychology 15(3): 337-351.

44. Hirdes JP, Scott KA (1998) Social relations in a chronic care hospital: a whole network study of patients, family and employees. Social Networks 20(2): 119-133.

45. Wright ER, Shuff IM (1995) Specifying the integration of mental health and primary health care services for persons with HIV/AIDS: The Indiana integration of care project. Social Networks 17(319-340.

46. Parchman ML, Scoglio CM, Schumm P (2011) Understanding the implementation of evidence-based care: a structural network approach. Implementation Science 6(14.

47. Goodwin N (2010) It's good to talk: social network analysis as a method for judging the strength of integrated care. International Journal of Integrated Care 10(e120.

48. Dunn AG, Westbrook JI (2011) Interpreting social network metrics in healthcare organisations: a review and guide to validating small networks. Social Science & Medicine 72(7): 1064-1068.

49. Malin B, Nyemba S, Paulett J (2011) Learning relational policies from electronic health record access logs. Journal of Biomedical Informatics 44(2): 333-342.

50. Hripcsak G, Vawdrey DK, Fred MR, Bostwick SB (2011) Use of electronic clinical documentation: time spent and team interactions. Journal of the American Medical Informatics Association 18(2): 112-117.

51. Effken JA, Benham-Hutchins M (2011) Technology-enhanced social network analysis: an odd idea whose time has come-again. Online Journal of Nursing Informatics 15(1): 326. Available: <http://ojni.org/issues/?p=326>.

52. Guldbrandsson K, Nordvik MK, Bremberg S (2010) Opinion leaders in child health promotion identified by network analysis, Sweden 2008. European Journal of Public Health 20(Suppl. 1)(246.

53. Varda DM (2011) Data-driven management strategies in public health collaboratives. Journal of Public Health Management and Practice 17(2): 122-132.

54. Christakis NA, Fowler JH (2011) Contagion in prescribing behavior among networks of doctors. Marketing Science 30(2): 213-216.

55. Palinkas LA, Holloway IW, Rice E, Fuentes D, Wu Q, et al. (2011) Social networks and implementation of evidence-based practices in public youth-serving systems: a mixed methods study. Implementation Science 6(113.

56. Doumit G, Wright FC, Graham ID, Smith A, Grimshaw J (2011) Opinion leaders and changes over time: a survey. Implementation Science 6(117.
