# Supplementary material for: Social Network Analysis in Healthcare Settings: A Systematic Scoping Review
Source: PLoS One. 2012 Aug 3;7(8):e41911. doi: 10.1371/journal.pone.0041911 (PMC3411695; doi:10.1371/journal.pone.0041911)
Supplement: File S1 — Review protocol. (DOCX) [file pone.0041911.s003.docx]

Final protocol: November 2010

**Social network analysis for quality improvement in healthcare: a systematic review**

**Background**

The TRiP-LaB research protocol includes the use of social network analysis (SNA) to explore communication networks in TRiP-LaB’s NHS partner organisations. The objective of this analysis is to understand the local context as part of the process of developing an implementation strategy. SNA can also be used as part of the process of implementation, e.g. by identifying ‘opinion leaders’. There appear to be no existing systematic reviews on the use of SNA in healthcare settings. A review of the current evidence base could be useful for both the TRiP-LaB team and the broader field of implementation research.

**Objectives**

Our primary objective is to evaluate the use of SNA as part of an intervention to support the implementation of change in healthcare organisations. A secondary objective is to identify and describe studies that report the results of an SNA in a healthcare setting: what do they tell us about the role and influence of social networks in healthcare organisations?

**Inclusion/exclusion criteria**

*Participants*

Healthcare professionals (e.g. doctors, nurses, pharmacists, radiographers etc. etc.) and others involved in their professional social networks (e.g. administrative, support and secretarial staff) in any healthcare setting.

*Intervention*

SNA maps and measures formal and informal relationships within organisations or other defined groups to understand what facilitates or hinders the flow of knowledge and information between individuals and groups. Data are normally gathered by questionnaires and/or interviews and analysed using SNA software.

To be eligible for the review, studies will have to describe and report the results of an SNA performed in a healthcare setting. Those that go on to report on the use of the results of the SNA as part of an intervention to change some aspect of policy or practice will be classified as level I studies. Studies that describe the existing social networks in the organisation without reporting any follow-up action or its results will be classified as level II studies.

*Comparators*

Eligible comparisons for level I studies are between organisations (SNA performed and used vs. no SNA) or within an organisation (before vs. after SNA performed and used). Level II studies need not have a comparator.

*Outcomes*

Any measure of the performance of a healthcare organisation or of individuals within it. We will distinguish between outcomes related to patient care (patient outcomes or process of care measures; primary outcomes) and broader measures of organisational performance (e.g. staff job satisfaction, staff retention or profitability of a private practice). Studies that use changes to social networks (measured by a follow-up SNA) as outcome measures will also be eligible. Level II studies can have properties of the social network as outcomes.

*Study designs*

Randomised and non-randomised controlled trials, controlled before and after studies and interrupted time series studies will be eligible for inclusion as level I studies. Level II studies may be of any design.

*Exclusion criteria*

Studies that use questionnaires or interviews to identify ‘opinion leaders’ but do not conduct an SNA will be excluded, as will studies of patients’ and carers’ social networks.

**Review methods**

*Searching*

We will search the following sources for published or unpublished studies in any language:

Databases: MEDLINE, EMBASE, PsycINFO, Health Management Information Consortium (HMIC), CINAHL, EBSCO Business Source Premier, Social Science Citation Index (SSCI), Conference Proceedings Citation Index: Social Science & Humanities, ASSIA, Cochrane Central Register of Controlled Trials (CENTRAL). Databases will be searched from inception or 1950, whichever is later.

We will also search the Cochrane Database of Systematic Reviews, DARE and the HTA database.

Supplementary searches of library and information science databases may be performed, depending on the results of the main searches.

Websites: We will search the International Network for Social Network Analysis ([www.insna.org](http://www.insna.org)) and other organisations active in the field. Websites of companies supplying SNA software will be searched for relevant unpublished case studies, although these will only be included if adequate data can be extracted.

Textbooks: We will screen the contents and reference lists of printed and online textbooks of SNA.

We will make contact with experts in the field of SNA (e.g. Thomas Valente, Rob Cross, Steve Borgatti) before finalising the list of sources to be searched.

*Study selection*

Search results will be stored in a reference management database. Two reviewers will independently assess titles and abstracts for relevance. Full-text copies of study reports will be obtained for items thought to be potentially relevant. A final decision on inclusion will be made by two reviewers independently using the inclusion criteria above. Disagreements between reviewers at either stage will be resolved by discussion or by reference to a third reviewer if necessary.

*Data extraction*

Data will be extracted by one reviewer and checked by another. Data extraction forms will be developed and tested on a small sample of studies if necessary. Details to be extracted where available will include:

Bibliographic details

Study design (RCT, CCT, CBA, ITS)

Study objective(s)

Unit of allocation

Participants (health professionals)

- Profession
- Clinical specialty
- Level of training
- Age
- Years since graduation or in practice
- Proportion of eligible professionals (or practices etc.) that participated in the study

Participants (patients)

- Clinical problem
- Age
- Gender
- Ethnicity

Numbers included in study (episodes of care, patients, health professionals, practices etc. as appropriate)

Setting

- Type of healthcare system
- Setting (e.g. inpatient, outpatient, general practice)
- Country

SNA intervention

- Objective(s) of the intervention (if not covered above)
- Type of SNA used
- Are details available (e.g. copy of questionnaire)?
- How many people received the questionnaire?
- How were participants selected?
- Response rate
- How were results used? (Results must be used if study is to meet inclusion criteria for level I evidence)

Other implementation intervention

- Type of intervention (e.g. reminders, audit and feedback, educational meetings, educational outreach, multifaceted)
- Objectives of intervention (if not covered above)
- How many people received the intervention?
- Brief details of intervention as applicable, e.g. group or individual, deliverer, timing, setting

Control group

- What did they receive? No intervention, standard care/practice or another intervention (specify)
- Number in control group

Results

We will extract study results for patient or process of care outcomes into tables (see draft tables below)

Studies with control group

| Study details | Study design | Comparison | Results |
| --- | --- | --- | --- |
|  |  |  | Outcome:  Pre-intervention: SNA group/control  Post-intervention: SNA group/control  Difference between post-intervention SNA and control:  Significance: |
|  |  |  |  |

ITS studies

| Study details | Comparison | Results |
| --- | --- | --- |
|  |  | Outcome:  Number of data points  Pre-intervention:  Post-intervention  Interval between data points:  Pre-intervention mean:  Post-intervention mean:  Difference between pre- and post-intervention means:  Significance: |
|  |  |  |

Results of studies that describe social networks only (level II) will be briefly summarised using free text.

*Quality assessment*

We will assess risk of bias in included studies using the Cochrane EPOC group criteria where applicable. Assessments will be performed by two reviewers independently. Any disagreements will be resolved by discussion, with arbitration by a third reviewer if necessary.

*Data synthesis*

We anticipate that heterogeneity of settings, interventions and outcomes will preclude meta-analysis. We will present a narrative synthesis of the included studies by type of outcome (patient care-related vs. other) and type of study design. Level I and level II studies will be considered separately. The synthesis will emphasise evidence from better quality studies and those in which SNA was a major component of the intervention.

*Dissemination*

We will aim to present the results at an appropriate conference(s) and publish a paper in an appropriate journal. Results will also be disseminated via TRiP-LaB (e.g. web site, newsletter).
