# Supplementary material for: Social Network Analysis in Healthcare Settings: A Systematic Scoping Review
Source: PLoS One. 2012 Aug 3;7(8):e41911. doi: 10.1371/journal.pone.0041911 (PMC3411695; doi:10.1371/journal.pone.0041911)
Supplement: File S3 — Details of search strategy. (DOCX) [file pone.0041911.s005.docx]

**Supporting File 3**

**Literature search strategy**

| **MEDLINE and MEDLINE In-Process & Other Non-Indexed Citations**  1948 – 30^th^ December 2010  OvidSP interface <http://ovidsp.ovid.com/>  Searched: 4^th^ January 2011  Records identified: 1692 |
| --- |
| 1 exp Interprofessional Relations/ (44247)  2 (network or networks).ti,ab. (160635)  3 1 and 2 (780)  4 ((social network or social networks) adj3 (analy$ or investigat$ or examin$ or explor$ or inquir$)).ti,ab. (410)  5 ((social network or social networks) adj3 (theor$ or framework$ or model$ or approach$ or pattern$ or perspective$)).ti,ab. (188)  6 ((social network or social networks) adj3 (method$ or technique$ or tool$ or survey$ or questionnaire$ or diagram$ or software or data or dataset$ or measur$ or metric$ or indicator$ or information)).ti,ab. (312)  7 ((social network or social networks) adj3 (intervention$ or stud$)).ti,ab. (179)  8 Sociometric Techniques/ (947)  9 (sociometr$ or sociogram$ or sociomap$).ti,ab. (559)  10 opinion leader$.ti,ab. (601)  11 8 or 9 or 10 (1908)  12 11 and 2 (112)  13 UCINET.ti,ab. (12)  14 NetDraw.ti,ab. (4)  15 Pajek.ti,ab. (9)  16 KrackPlot.ti,ab. (1)  17 3 or 4 or 5 or 6 or 7 or 12 or 13 or 14 or 15 or 16 (1719)  18 exp animals/ not humans.sh. (3471914)  19 17 not 18 (1692)  20 limit 19 to yr="1950 -Current" (1692) |
| **Key**  / = indexing term (MeSH heading)  exp = exploded MeSH heading  $ = truncation  .ti,ab. = terms in either title or abstract fields  adj3 = terms within three words of each other (any order)  .sh.= subject heading field |

The MEDLINE search strategy above was adapted for use in the following databases:

- EMBASE (via OvidSP, 1980 – 2010 week 52, searched: 4^th^ January 2011)
- PsycINFO (via OvidSP, 1806 – January week 1 2011, search: 6^th^ January 2011)
- Health Management Information Consortium (HMIC) (via OvidSP, inception – November 2010, searched: 4^th^ January 2011)
- The Cochrane Library, via Wiley (all searched: 22^nd^ December 2010)
  - Cochrane Database of Systematic Reviews (CDSR) (Issue 12, 2010)
  - Database of Abstracts of Reviews of Effects (DARE) (Issue 4, 2010)
  - Cochrane Central Register of Controlled Trials (CENTRAL) (Issue 4, 2010)
  - Cochrane Methodology Register (CMR) (Issue 4, 2010)
  - Health Technology Assessment Database (HTA) (Issue 4, 2010)
- Cumulative Index to Nursing & Allied Health (CINAHL) (via EBSCO, 1980 – 10^th^ December 2010, searched: 7^th^ January 2011)
- Business Source Premier (via EBSCO, inception – 9^th^ January 2011, searched: 10^th^ January 2011)
- Social Science Citation Index (via ISI Web of Knowledge, 1956 – present, searched: 7^th^ January 2011)
- Conference Proceedings Citation Index- Social Science & Humanities (via ISI Web of Knowledge, 1990 – present, searched: 7^th^ January 2011)
- ASSIA (via CSA Illumina, 1987 – December 2010, searched: 10^th^ January 2011)

Searches for all databases were re-run in October 2011 to capture more recently published studies. The full search strategies for each database are available on request from the authors.
